# Outcomes and predictors of progression in progressive pulmonary fibrosis

**DOI:** 10.1080/07853890.2024.2406439

**Published:** 2024-09-23

**Authors:** Zekai Cen, Tiantian Cen, Qunli Ding, Yun Zhang, Pan Tang, Chengna Lv, Tingting Wu

**Affiliations:** Department of Respiratory and Critical Care Medicine, Key Laboratory of Respiratory Disease of Ningbo, The First Affiliated Hospital of Ningbo University, Ningbo, Zhejiang, China

**Keywords:** Progressive pulmonary fibrosis, interstitial lung disease, tumour markers, acute exacerbation

## Abstract

**Background:**

Progressive pulmonary fibrosis (PPF) is a general term for a class of interstitial lung diseases (ILDs) characterized by a progressive fibrosing (PF) phenotype. Patients with PPF have decreased lung function, exercise ability, and quality of life. The purpose of this study was to investigate the clinical characteristics, potential associated factors for disease progression, and survival outcomes of patients in the PPF population.

**Methods:**

This study retrospectively reviewed the data of patients diagnosed with ILD between January 2011 and December 2022 at The First Affiliated Hospital of Ningbo University. A PF phenotype was defined based on the criteria that were used in the PPF clinical practice guidelines, which led to the identification of 92 patients with a PF phenotype among the 177 patients with fibrotic ILD. Baseline clinical information and laboratory parameters were collected and analysed in our cohort.

**Results:**

Patients in the PPF group had higher tumour marker levels and lower pulmonary function test results at baseline than did those in the non-PPF group. According to the multivariate logistic regression analysis, age >65 years (OR 2.71, 95% CI 1.26-5.89; *p* = 0.011), LDH >245 U/L (OR 3.07, 95% CI 1.39-6.78; *p* = 0.006), CA-153 > 35 U/mL (OR 3.16, 95% CI 1.25-7.97; *p* = 0.015), FVC <60% predicted (OR 4.82, 95% CI 1.60-14.51; *p* = 0.005), DLCO <50% predicted (OR 3.21, 95% CI 1.43-7.21; *p* = 0.005), and the UIP-like pattern on chest HRCT (OR 3.65, 95% CI 1.33-10.07; *p* = 0.012) were potentially associated with the progression of fibrotic interstitial lung diseases (f-ILDs) to PPF. Furthermore, the PPF group had a poorer survival rate than the non-PPF group (*p* = 0.0045). According to the multivariate Cox regression analysis, an SPAP ≥ 37 mmHg (HR 2.33, 95% CI 1.09-5.00; *p* = 0.030) and acute exacerbation (HR 2.88, 95% CI 1.26-6.59; *p* = 0.012) were identified as significant prognostic factors for mortality in patients with PPFs.

**Conclusions:**

Patients who were older, had high CA-153 and LDH levels, had poor pulmonary function test results, or had a UIP-like pattern on chest HRCT were more likely to have indications for the progression of f-ILD to PPF. Increased SPAP and AE are independent risk factors for the prognosis of PPF patients, so additional attention should be given to such patients.

## 1. Introduction

Progressive pulmonary fibrosis (PPF) is a new term used to describe the condition that represents the progression of pulmonary fibrosis despite appropriate treatment. Idiopathic pulmonary fibrosis (IPF), the most common, specific, severe phenotype of idiopathic interstitial pneumonia (IIP), is a chronic fibrotic interstitial lung disease with irreversible progression [1–5]. In addition to IPF, all of the subtypes of any ILD subtype may develop into progressive pulmonary fibrosis despite current therapy [6–9]. These conditions may have similar pathology and clinical behaviours; therefore, the new concept of ‘progressive pulmonary fibrosis (PPF)’ has been proposed for patients with a fibrotic interstitial lung disease (f-ILD) with a progressive fibrosing (PF) phenotype in recent years.

A reduction in the forced vital capacity (FVC), a reduction in the diffusing capacity of the lung for carbon monoxide (DLCO), worsening respiratory symptoms, and increased fibrosis on high-resolution computed tomography (HRCT) are usually used to assess patients with PPF [8,10,11]. Previous studies have shown that 18-32% of patients diagnosed with non-IPF ILDs develop PPF. Clinicians have estimated that the time from ILD diagnosis to the development of PPF is 11-15 months. However, the estimated time between the detection of a PF phenotype and death has been reported to be 30-45 months, which is similar to that of IPF [12]. The delay in the diagnosis of PPF accompanied by the delay in antifibrotic therapy may be the main reason for the poor prognosis of patients with this disease. Therefore, the use of serum biomarkers and other indices to identify individuals at risk of PPF requires further investigation to guide the early initiation of antifibrotic therapy to improve the survival of these patients. In recent years, some biomarkers have been shown to be potentially related to the progression or prognosis of PPF, such as KL-6 and various miRNAs [13,14].

In this single-centre cohort study, we retrospectively analysed the baseline clinical data of patients who were diagnosed with fibrotic ILD (except for IPF) in the past twelve years to explore whether there were clinical indicators associated with the progression of fibrotic ILD at an early stage. In addition, we evaluated patient survival outcomes and identified prognostic risk factors in the PPF population.

## 2. Methods

### 2.1. Study design

We retrospectively reviewed the medical records of patients who were diagnosed with ILD, including IPF, idiopathic nonspecific interstitial pneumonia (iNSIP), connective tissue disease (CTD)-associated ILD, fibrotic hypersensitivity pneumonitis (FHP), unclassifiable ILD, and ILD related to occupational exposures, between January 2011 and December 2022 at The First Affiliated Hospital of Ningbo University. The first diagnosis of PPF was in July 2015. Each disease was diagnosed according to its respective criteria [15–26].

Disease progression was evaluated at each hospital visit by using a time window of 12 months before each hospital visit until the first event, which met the definition criteria for progression, was confirmed. The selected patients had features of fibrosing lung disease on high-resolution CT. In particular, when considering the various definitions of PPF in different studies [6,9,27–29], we adopted the following inclusion criteria based on the PPF clinical practice guidelines to assess disease progression within the 12 months before screening [29].

Patients who met the diagnostic criteria for PPF were ultimately included in the PPF group. On the other hand, the non-PPF group included patients with ILD (other than IPF) who had pulmonary fibrosis on high-resolution CT but could not be included in the PPF group. Patients diagnosed with IPF were included in a separate group [2].

### 2.2. Data collection

The clinical data, including detailed patient histories, clinical manifestations, laboratory results, and chest HRCT, were retrospectively obtained from the medical records and telephone interviews. The data included basic patient information (age, sex, medical history, diagnosis, smoking status, and occupational history) and laboratory data (arterial blood gas analysis, lactate dehydrogenase, erythrocyte sedimentation rate, alanine aminotransferase, aspartate aminotransferase, creatine kinase, C-reactive protein, D-dimer, carcinoembryonic antigen (CEA), and serum tumour markers). The cut-off values of laboratory results in logistic regression analysis and survival analysis were defined based on the reference range in our hospital’s laboratory test form. For example, the reference range for CEA is listed as 0 to 5 μg/L, and so a value greater than the upper limit of this range this indicates an increase in the detection result.

Pulmonary function test (PFT) data, including FVC% predicted, FEV1, FEV1/FVC, DLCO% predicted, and subsequent hospitalization or outpatient PFTs, were also obtained. Furthermore, echocardiographic estimates of systolic pulmonary artery pressure (SPAP) were also recorded. Radiographic information, including all previous chest CT (computed tomography) and HRCT scans, was collected. Independent, separate, retrospective reviews of the chest HRCT images of each patient were performed by two professional and experienced thoracic radiologists. Two thoracic radiologists compared the chest HRCT images side by side to assess progression visually and determine the presence of pulmonary fibrosis. The definitions of acute exacerbation of ILD included acute worsening or development of dyspnoea < 1 month in duration, chest HRCT with new bilateral ground-glass opacities and/or consolidation superimposed on fibrosis, and deterioration that was not fully explained by cardiac failure or fluid overload [30,31]. The ILD patterns were classified according to the criteria of the 2013 ATS/ESR classification of idiopathic interstitial pneumonia (IIPs) and the usual interstitial pneumonia (UIP) pattern according to the criteria of the 2018 ATS/ERS/JRS/ALAT collaboration to develop a clinical practice guideline for the diagnosis and management of IPF [2,15]. The last follow-up was in December 2022. This study was approved by the Ethics Committee of The First Affiliated Hospital of Ningbo University (IRB no: 2024-003RS), and all of the methods were performed in accordance with relevant guidelines and regulations.

### 2.3. Statistical analysis

The statistical analysis was performed using IBM SPSS 26 and GraphPad Prism 9 software. Continuous normally distributed variables are presented as the mean ± standard deviation (SD), and nonnormally distributed variables are presented as the median and interquartile range. For two-group comparisons of binary data, the chi-square test was used. The t test or Mann–Whitney U test was used to compare normally distributed variables between the 2 groups, and nonnormally distributed variables were tested by using the Wilcoxon rank sum test. Univariate logistic regression analysis was used to identify factors associated with the PPF and non-PPF groups, and variables with *p* < 0.2 were included in the multivariate logistic regression model by using the backwards likelihood ratio test. Kaplan–Meier survival analysis was performed to estimate the survival rates and plot survival curves, and the log-rank test was used to compare the intergroup differences. Risk factors for mortality in the PPF group were identified by univariate analysis, and variables with *p* < 0.2 were included in the multivariate Cox regression model by using the forward likelihood ratio test. *p* < 0.05 was considered to indicate statistical significance.

## 3. Results

### 3.1. Selected population

**Figure 1.**
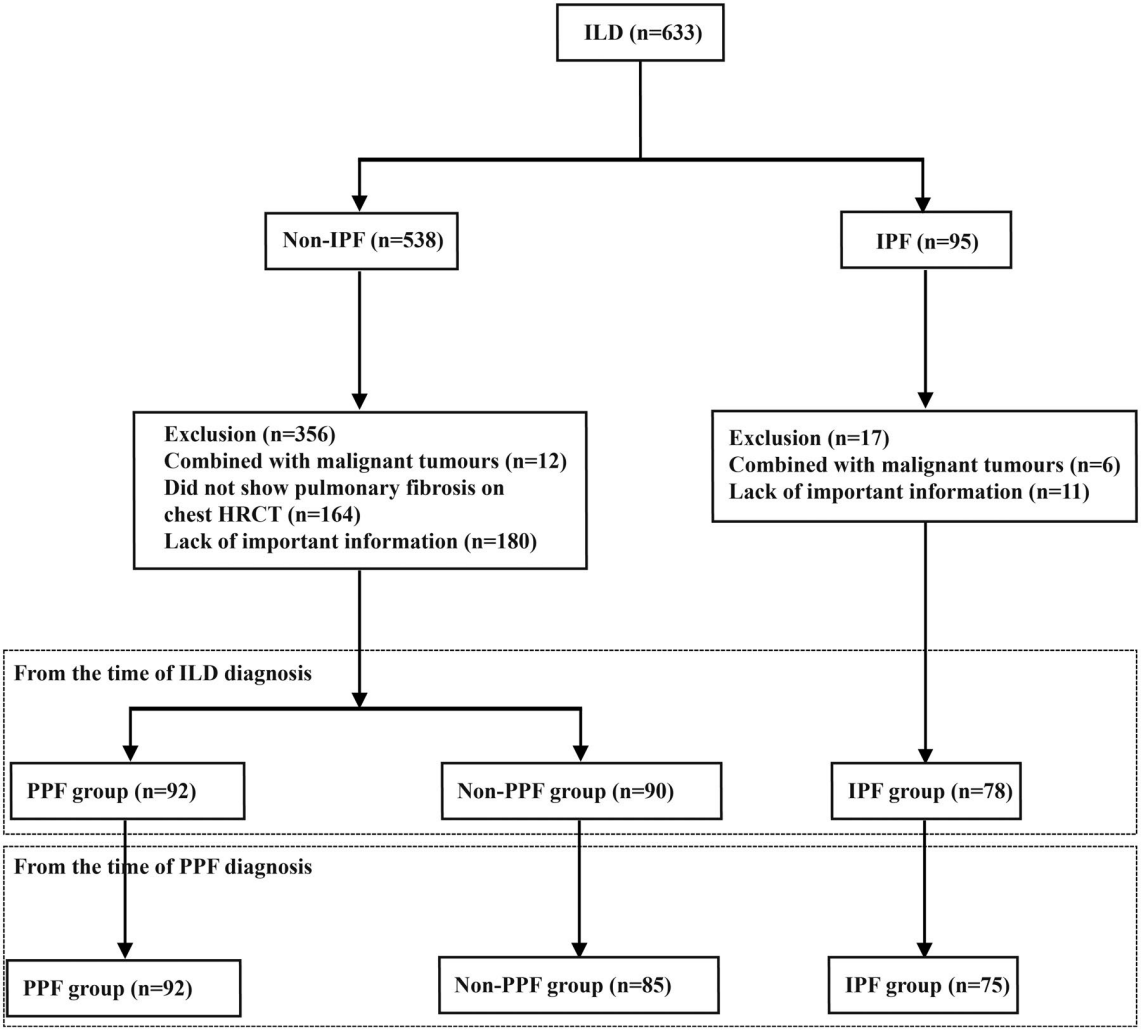
Patient recruitment flowchart. ILD: interstitial lung disease; PPF: progressive pulmonary fibrosis; IPF: idiopathic pulmonary fibrosis.

Five hundred thirty-eight patients diagnosed with ILD (other than IPF) were screened (Figure 1). A total of 356 patients were excluded: 12 patients had lung cancer, 164 patients did not have pulmonary fibrosis on chest HRCT, and 180 patients with pulmonary fibrosis were excluded due to a lack of important clinical information, such as PFTs. Finally, 177 patients who were assessed for eligibility were divided into two groups according to the inclusion criteria: 92 patients who were identified to have a progressive fibrosing phenotype were included in the PPF group, and 85 patients met the requirements of the non-PPF group. Among the patients who met the PPF criteria, 34 (37.0%) had worsening respiratory symptoms and physiological evidence of disease progression, 31 (33.7%) had worsening respiratory symptoms and radiological evidence of disease progression, and 27 (29.3%) had physiological evidence of disease progression and radiological evidence of disease progression.

In the PPF cohort, which included 51 patients with CTD-ILDs, 14 patients with ILD with antineutrophil cytoplasm antibody (ANCA)-associated vasculitis (AAV-ILD), 13 patients with IPAF, 9 patients with iNSIP, and 4 patients with ILDs related to occupational exposure, as well as CTD-ILDs alone, the proportion of patients with polymyositis/dermatomyositis-associated ILD was the largest (39.2%). Eighty-five patients with pulmonary fibrosis on HRCT who could not be diagnosed with PPF were included in the non-PPF group. Almost half of the patients in the two groups had been treated with corticosteroids combined with immunosuppressants. The detailed inclusion criteria for the study population are shown in Figure 1 and Table 1.

**Table 1.** Characteristics of the PPF group and non-PPF group.

Subtype	PPF group (N, %)	Non-PPF group (N, %)
CTD-ILDs	51 (55.4%)	65 (76.5%)
RA-ILD	15	12
pSS-ILD	9	12
SSc-ILD	5	16
PM/DM-ILD	20	12
SLE-ILD	1	6
MCTD-ILD	1	7
IPAF	13 (14.1%)	8 (9.4%)
AAV-ILD	14 (15.2%)	3 (3.5%)
FHP	1 (1.1%)	0 (0%)
iNSIP	9 (9.8%)	6 (7.1%)
ILDs related to occupational exposures	4 (4.3%)	0 (0%)
Unclassifiable ILD	0 (0%)	3 (3.5%)
Total	92	85
Initial treatment		
Corticosteroids alone	23 (25.0%)	21 (24.7%)
Corticosteroids + immunosuppressants	46 (50.0%)	47 (55.3%)
Antifibrotic agents	30 (32.6%)	8 (9.4%)
Biological agents	1 (1.1%)	8 (9.4%)
Only immunosuppressants	11 (12.0%)	3 (3.5%)
No treatment	12 (13.0%)	14 (16.5%)
Acute exacerbation		
At least one acute exacerbation	39 (42.4%)	2 (2.4%)

PPF: progressive pulmonary fibrosis; ILD: interstitial lung disease; CTD: connective tissue disease; RA: rheumatoid arthritis; pSS: primary Sjögren’s syndrome; SSc: systemic sclerosis; PM/DM: polymyositis/dermatomyositis; SLE: systemic lupus erythematosus; MCTD: mixed connective tissue disease; IPAF: interstitial pneumonia with autoimmune features; AAV: antineutrophil cytoplasm antibody (ANCA)-associated vasculitis; FHP: fibrotic hypersensitivity pneumonitis; iNSIP: idiopathic nonspecific interstitial pneumonia; ILDs related to occupational exposures: including asbestosis and silicosis.

### 3.2. Clinical characteristics

A comparison of the clinical characteristics of the PPF group and the non-PPF group is shown in Table 2. The mean age of the patients in the PPF group was 66.14 ± 10.95 years (interquartile range [IQR], 60.25-73.00 years), and the ratio of males to females was 1:1.09. Age, sex, and smoking status were significantly different between the PPF group and the non-PPF group.

**Table 2.** Comparison of the clinical characteristics between the PPF group and the non-PPF group.

Factors	PPF(*n* = 92)	Non PPF(*n* = 85)	*P-*value
Age (year)	66.14 ± 10.95 [60.25-73.00]	60.07 ± 13.13 [49.50-70.00]	0.001
M/F	44/48	23/62	0.004
Smoking	31 (33.7%)	14 (16.5%)	0.009
Drinking	19 (20.7%)	10 (11.8%)	0.233
OI (mmHg)^1^	363.28 ± 82.25 (*n* = 86)	379.16 ± 69.27 (*n* = 53)	0.243
P(A-a)O_2_ (mmHg)	36.72 ± 32.58 (*n* = 86)	28.71 ± 22.37 (*n* = 53)	0.088
LDH (U/L)	265.90 ± 103.17	232.47 ± 95.64	0.027
ESR (mm/h)	49.61 ± 30.91	37.33 ± 25.67	0.005
ALT (U/L)	22.59 ± 25.61	23.71 ± 23.48	0.813
AST (U/L)	29.59 ± 30.96	28.67 ± 18.32	0.763
CK (U/L)	161.38 ± 449.08	103.64 ± 151.73	0.247
CRP (mg/L)	24.90 ± 47.56	13.11 ± 25.36	0.039
SPAP (mmHg)	29.65 ± 15.02	23.86 ± 11.89	0.005
D-Dimer (µg/L)	299.27 ± 269.93	349.70 ± 507.14	0.416
AFP (µg/L)	2.423 ± 1.086	2.601 ± 1.280	0.319
CEA (µg/L)	7.991 ± 36.650	2.764 ± 2.339	0.176
CA-199 (U/mL)	63.02 ± 129.57	15.01 ± 24.43	0.001
CA-125 (U/mL)	38.85 ± 43.36	24.01 ± 64.03	<0.001
CA-153 (U/mL)	46.91 ± 66.81	20.63 ± 20.15	<0.001
FVC (% predicted)	67.31 ± 15.81	86.37 ± 16.83	<0.001
FEV1 (% predicted)	72.57 ± 18.48	88.93 ± 18.59	<0.001
FEV1/FVC (%)	86.35 ± 7.74	84.15 ± 7.12	0.073
DLCO (% predicted)	48.48 ± 18.63	63.45 ± 17.61	<0.001
UIP-like Pattern	31(33.7%)	9 (10.6%)	<0.001
AE	37 (40.2%)	2 (2.4%)	<0.001

The data are presented as the mean ± SD, median (IQR) or no. (%), and *p* < 0.05 was considered to indicate statistical significance.

PPF: progressive pulmonary fibrosis; M/F: male/female; OI: oxygenation index; P(A-a)O2: alveolar-arterial oxygen gradient; LDH: lactate dehydrogenase; ESR: erythrocyte sedimentation rate; ALT: alanine aminotransferase; AST: aspartate aminotransferase; CK: creatine kinase; CRP: C-reactive protein; SPAP: systolic pulmonary artery pressure; AFP: alpha-fetoprotein; CEA: carcinoembryonic antigen; CA-199: carbohydrate antigen 199; CA-125: carbohydrate antigen 125; CA-153: carbohydrate antigen 153; FVC: forced vital capacity; FEV1: forced expiratory volume in one second; FEV1/FVC: forced vital capacity rate of one second; DLCO: diffusing capacity of the lung for carbon monoxide; UIP: usual interstitial pneumonia; and AE: acute exacerbation.

**^1^** Arterial blood gas analysis (including OI and P(A-a)O_2_) was not performed for 6 patients in the PPF group or 32 in the non-PPF group.

Among the serum biomarkers, high levels of lactate dehydrogenase (LDH), erythrocyte sedimentation rate (ESR), C-reactive protein (CRP), carcinoembryonic antigen (CEA), carbohydrate antigen 199 (CA-199), carbohydrate antigen 125 (CA-125), and carbohydrate antigen 153 (CA-153) were observed in the PPF group, and these levels were significantly different from those in the non-PPF group. The PFTs in the PPF group had worse disease than did those in the non-PPF group, as indicated by the FVC % predicted, FEV1% predicted and DLCO % predicted. In addition, compared with those in the non-PPF group, there was a greater proportion of patients with high SPAP and UIP-like patterns on chest HRCT and at least one acute exacerbation in the PPF group.

### 3.3. Factors associated with the development of PPF

The logistic regression analysis, which was used to identify factors associated with developing PPF, is shown in Table 3. Univariate logistic regression analysis indicated that age >65 years, male sex, LDH >245 U/L, CRP >10 mg/L, CEA >5 µg/L, CA-199 > 35 U/mL, CA-125 > 35 U/mL, CA-153 > 35 U/mL, FVC <60% predicted, DLCO <50% predicted and UIP-like pattern on chest HRCT were all potential factors associated with the progression of f-ILD to PPF. However, in the multivariate logistic regression analysis, age >65 years (OR 2.71, 95% CI 1.26-5.89; *p* = 0.011), LDH >245 U/L (OR 3.07, 95% CI 1.39-6.78; *p* = 0.006), CA-153 > 35 U/mL (OR 3.16, 95% CI 1.25-7.97; *p* = 0.015), FVC <60% predicted (OR 4.82, 95% CI 1.60-14.51; *p* = 0.005), DLCO <50% predicted (OR 3.21, 95% CI 1.43-7.21; *p* = 0.005) and UIP-like pattern on chest HRCT (OR 3.65, 95% CI 1.33-10.07; *p* = 0.012) were considered potential associated factors for developing PPF.

**Table 3.** Factors associated with developing PPF based on logistic regression.

Factors	Univariate		Multivariate	
	OR (95% CI)	*P-*value	OR (95% CI)	*P-*value
Age >65 (year)	2.37 (1.29-4.34)	0.005	2.71 (1.26-5.89)	0.011
Male	2.47 (1.32-4.64)	0.005	—	NS
LDH >245 (U/L)	3.07 (1.64-5.77)	<0.001	3.07 (1.39-6.78)	0.006
ESR >60 (mm/h)	1.84 (0.90-3.77)	0.097	—	NS
ALT >40 (U/L)	1.04 (0.38-2.84)	0.933		
AST >40 (U/L)	0.91 (0.39-2.16)	0.835		
SPAP ≥ 37 (mmHg)	2.15 (1.00-4.63)	0.051	—	NS
CRP >10 (mg/L)	1.96 (1.03-3.74)	0.042	—	NS
CEA >5 (µg/L)	2.37 (1.08-5.21)	0.031	—	NS
CA-199 > 35 (U/mL)	4.89 (2.10-11.41)	<0.001	—	NS
CA-125 > 35 (U/mL)	6.06 (2.37-15.51)	<0.001	—	NS
CA-153 > 35 (U/mL)	4.40 (2.01-9.63)	<0.001	3.16 (1.25-7.97)	0.015
FVC <60 (% predicted)	7.34 (3.45-15.60)	<0.001	4.82 (1.60-14.51)	0.005
DLCO <50 (% predicted)	3.99 (2.13-7.47)	<0.001	3.21 (1.43-7.21)	0.005
UIP-like Pattern	4.29 (1.90-9.70)	<0.001	3.65 (1.33-10.07)	0.012

*p* < 0.05 was considered to indicate statistical significance.

OR: odds ratio; 95% CI: 95% confidence interval; NS: not significant; LDH: lactate dehydrogenase; ESR: erythrocyte sedimentation rate; ALT: alanine aminotransferase; AST: aspartate aminotransferase; SPAP: systolic pulmonary artery pressure; CRP: C-reactive protein; CEA: carcinoembryonic antigen; CA-199: carbohydrate antigen 199; CA-125: carbohydrate antigen 125; CA-153: carbohydrate antigen 153; FVC: forced vital capacity; DLCO: diffusing capacity of the lung for carbon monoxide; UIP: usual interstitial pneumonia.

### 3.4. Survival

During the follow-up, 64 patients died in all three groups, 29 died in the PPF group, and 4 died in the non-PPF group. The median durations of follow-up from the time of PPF diagnosis to the time of ILD diagnosis were 26.0 months (IQR 14.25-36.75 months) and 57.0 months (IQR 39.25-90.25 months), respectively. The median survival time from the diagnosis of disease progression with PPF was 58 months. The mean time from the diagnosis of ILD to the development of PPF was 34.76 ± 24.93 months. The 1-year, 3-year, and 5-year survival rates in the PPF group were 93.08%, 66.32%, and 37.44%, respectively.

**Figure 2.**
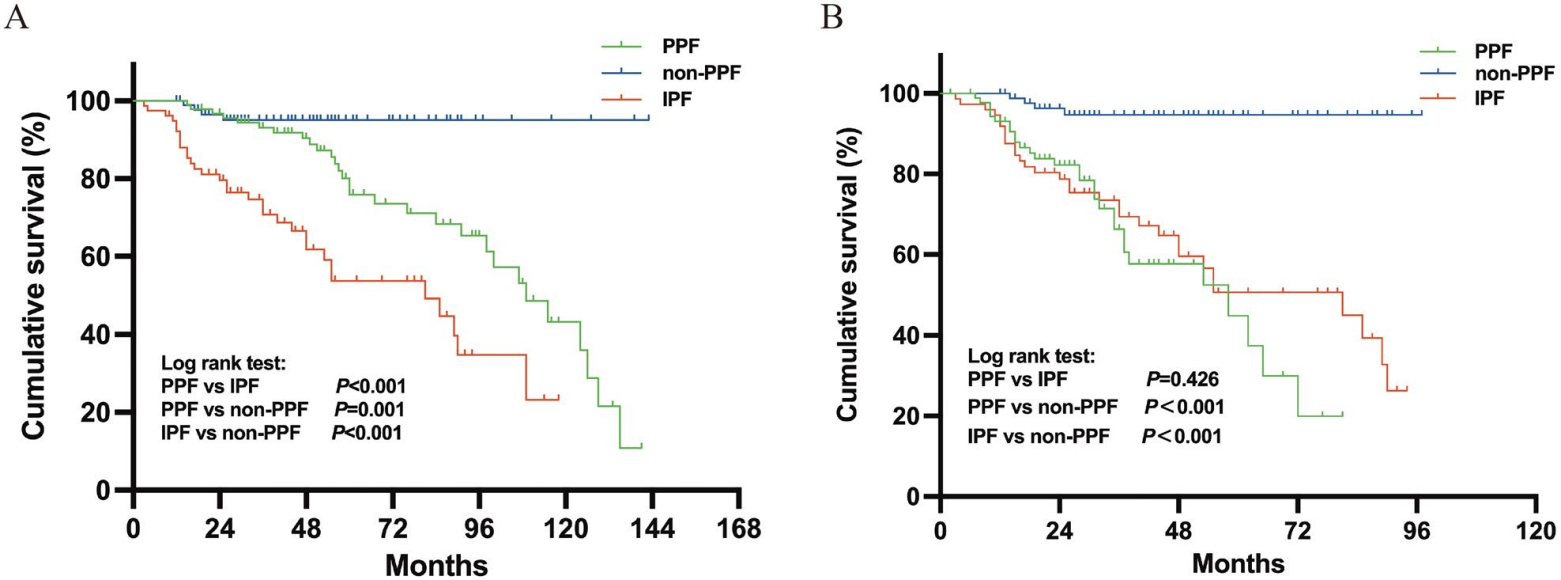
(A) Kaplan–Meier survival curves of the PPF (from the time of ILD diagnosis), non-PPF, and IPF groups. (B) Kaplan–Meier survival curves of the PPF (from the time of PPF diagnosis), non-PPF, and IPF groups. IPF: idiopathic pulmonary fibrosis; PPF: progressive pulmonary fibrosis.

Kaplan–Meier survival curves were generated to estimate the survival rate of each group from the first diagnosis of ILD, as shown in Figure 2A. The IPF group had a worse survival rate than did the PPF group (*p* < 0.001) and the non-PPF group (*p* < 0.001). After the diagnosis of ILD progression, the survival rate of the PPF group was similar to that of the IPF group, and there was no significant difference. However, there was a significant difference in the survival rate between the PPF group and the non-PPF group (*p* < 0.001) (Figure 2B).

**Figure 3.**
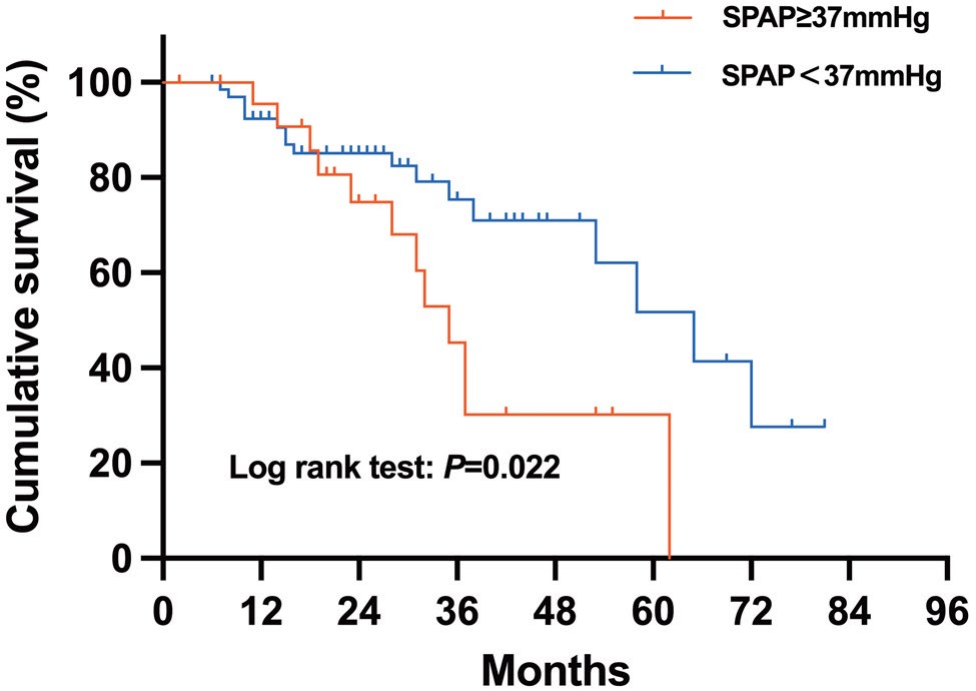
Kaplan–Meier survival curves of patients with a SPAP <37 mmHg and a SPAP ≥37 mmHg in the PPF group. SPAP: systolic pulmonary artery pressure; PPF: progressive pulmonary fibrosis.

**Figure 4.**
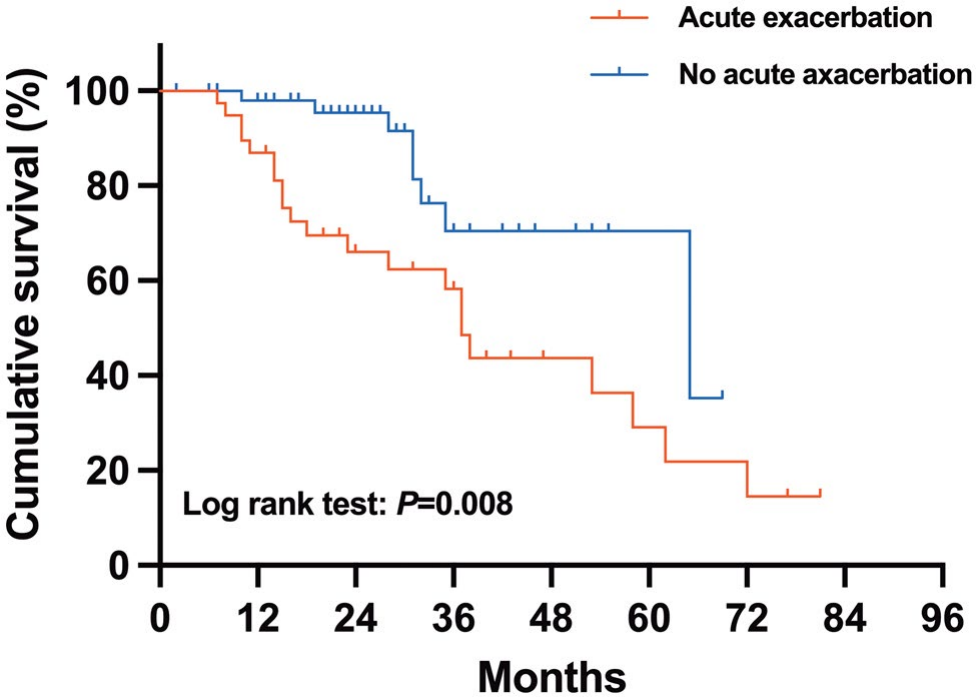
The Kaplan–Meier survival curves of patients who had at least one acute exacerbation and those who did not have any acute exacerbations in the PPF group.

Univariate and multivariate analyses of the risk of all-cause mortality in patients with PPF are summarized in Table 4. According to the univariate Cox analysis, survival was significantly shorter for patients who had LDH >245 U/L (hazard ratio (HR) 2.25, 95% CI 1.08-4.67; *p* = 0.030), SPAP ≥ 37 mmHg (HR 2.27, 95% CI 0.97-5.34; *p* = 0.022) (Figure 3) or who experienced acute exacerbation (HR 2.76, 95% CI 1.33-5.73; *p* = 0.008) (Figure 4). According to the multivariate Cox regression analysis, an SPAP ≥ 37 mmHg (HR 2.33, 95% CI 1.09-5.00; *p* = 0.030) and acute exacerbation (HR 2.88, 95% CI 1.26-6.59; *p* = 0.012) were found to be significant factors that were independently associated with shorter survival.

**Table 4.** Prognostic factors for mortality in patients with PPF (from the time of PPF diagnosis) based on Cox regression.

Factors	Univariate		Multivariate	
	HR (95% CI)	*P-*value	HR (95% CI)	*P-*value
Age >65 (years)	1.01 (0.48-2.15)	0.970		
Male	1.40 (0.68-2.90)	0.359		
LDH >245 (U/L)	2.25 (1.08-4.67)	0.030	—	NS
SPAP ≥ 37 (mmHg)	2.27 (0.97-5.34)	0.022	2.33 (1.09-5.00)	0.030
CRP >10 (mg/L)	0.53 (0.24-1.18)	0.118	—	NS
CA-199 > 35 (U/mL)	1.11 (0.53-2.32)	0.772		
CA-125 > 35 (U/mL)	1.30 (0.61-2.79)	0.473		
CA-153 > 35 (U/mL)	0.80 (0.37-1.71)	0.569		
FVC <60 (% predicted)	1.08 (0.51-2.28)	0.826		
DLCO <50 (% predicted)	1.63 (0.77-3.44)	0.233		
UIP-like Pattern	0.98 (0.46-2.11)	0.964		
AE	2.76 (1.33-5.73)	0.008	2.88 (1.26-6.59)	0.012
Antifibrotic agent	2.49 (1.11-5.57)	0.077	—	NS

*p* < 0.05 was considered to indicate statistical significance.

HR: hazard ratio; 95% CI: 95% confidence interval; NS: not significant; LDH: lactate dehydrogenase; SPAP: systolic pulmonary artery pressure; CRP: C-reactive protein; CA-199: carbohydrate antigen 199; CA-125: carbohydrate antigen 125; CA-153: carbohydrate antigen 153; FVC: forced vital capacity; DLCO: diffusing capacity of the lung for carbon monoxide; UIP: usual interstitial pneumonia; AE: acute exacerbation.

## 4. Discussion

In our present study, 92 out of 538 patients who were diagnosed with ILD other than IPF developed a progressive fibrosing phenotype, and age >65 years, LDH >245 U/L, CA-153 > 35 U/mL, FVC <60% predicted, DLCO <50% predicted, and the UIP-like pattern on chest HRCT were identified as potential associated factors for the progression from f-ILD to PPF. In addition, the IPF group had a worse survival rate than the PPF group (from the time of ILD diagnosis) and the non-PPF group. However, after the diagnosis of ILD progression, the PPF group had a similar poor survival rate as the IPF group. A SPAP ≥37 mmHg and acute exacerbation were identified as prognostic factors for mortality in the PPF population.

Some 14.5-32% of patients with non-IPF fibrotic ILD develop PPF, and the mean time from the first diagnosis of ILD to the development of PPF is 11-15 months [12,32,33]. In our study, the mean time from the first diagnosis of ILD to the development of PPF was 34.76 months. This is significantly longer than that reported in a previous study [12]. This was related to the irregular follow-up of ILD patients in the early stage and the failure of clinicians to pay more attention to ILD during the development of PPF. In addition, our present study showed that the IPF group had a worse survival rate than the PPF group from the first diagnosis of ILD, but after identification of a PF phenotype, the PPF group had a poor survival rate similar to that of IPF patients; therefore, more attention should be given to PPF patients, especially those with early-stage progression of f-ILD.

Our present study showed that age >65 years, LDH >245 U/L, CA-153 > 35 U/mL, FVC <60% predicted, DLCO <50% predicted, and the UIP-like pattern on chest HRCT were potential associated factors for developing PPF. Ageing is a driving force in the development of ILDs, such as RA-ILD and CHP [34,35]. Several scholars believe that abnormal telomere shortening and epithelial cell senescence may mechanistically underlie the progressive fibrotic phenotypes of various ILDs [36]. In general, the serum LDH concentration has been identified as a useful biomarker for disease activity and the severity of acute exacerbation in IPF [30,37,38]. Lang et al. [39] showed that elevated levels of serum LDH were associated with a more severe degree of pulmonary fibrosis in ILD patients, which was mainly related to hypoxia. Therefore, we hypothesized that a high LDH level may reflect the severity of pulmonary endogenous fibrosis and indicate disease progression in patients with IPF or other ILDs. On the other hand, there was a significantly high expression of serum tumour markers, such as CEA, CA-199, CA-125, and CA-153, in the PPF population. Maher et al. [40] reported that increased concentrations of CA19-9 were highly predictive of progressive fibrosis in IPF, and increasing concentrations of CA-125 predicted both disease progression and overall survival. High levels of serum tumour markers may also be associated with alveolar epithelial cell injury, possibly resulting in aggravated pulmonary endogenous fibrosis [41,42].

In addition, low baseline values of FVC % predicted and DLCO % predicted are often considered risk factors for the progression of PPF [43–46]. In a study of 135 patients with PPF, Kwon et al. [44] reported that fibrotic HP and a lower FVC were associated risk factors for PPF. In 132 patients with PPF, Chen et al. [45] reported that age >67 years, FVC % predicted from 40 to 59%, and DLCO % predicted <60% were risk factors for mortality in patients with PPF. Moreover, Zamora-Legoff et al. [46] also found that lower baseline values of DLCO % predicted and FVC % predicted increased the risk of progression in RA-ILD patients; additionally, in patients with unclassifiable ILD, DLCO has also been shown to be a predictor of disease progression and mortality [47]. Our present study also showed that patients with a UIP-like pattern on chest HRCT had more rapid disease progression. These results are consistent with previous research [48,49]. The UIP-like pattern on chest HRCT is also generally considered associated with worse outcomes, especially in patients with specific radiological features, such as honeycombing and bronchiectasis [10,50]. Wang et al. [51] showed that the UIP-like pattern on HRCT was associated with disease progression and worse mortality in patients with PF. However, our study did not reveal that the baseline FVC, baseline DLCO% or UIP-like pattern on chest HRCT were associated with poorer prognosis in PPF patients, which may be related to the high heterogeneity of the PPF and indicated that baseline PFT data and chest CT patterns cannot predict the prognosis of PPF well.

Our study revealed that an SPAP ≥ 37 mmHg was an independent risk factor for mortality in patients with PPF, as confirmed by Cox analysis. Previous research has indicated that the presence of pulmonary hypertension in the setting of ILD portends a poor prognosis and increased mortality rates [52]. Until now, echocardiography was a commonly used method for assessing SPAP, but there have been few reports on its relevance in the PPF cohort. In a retrospective study of 132 patients with PPF, an SPAP >36.5 mmHg was associated with mortality, which aligned with our findings [45]. Although right heart catheterization is the gold standard for the diagnosis of pulmonary hypertension, we emphasize the importance of screening and monitoring the SPAP by echocardiography during follow-up in patients with PPF.

Acute exacerbation is the most extreme form of IPF and the leading cause of death in patients with IPF [53]. Accumulating evidence indicates that patients with ILDs other than IPF also develop AE during their clinical course and have an extremely poor prognosis [54,55], and the highest AE rates have been reported in patients with a histological or radiological pattern of UIP [53–55]. In our study, patients in the PPF group with at least one AE had a poorer survival rate than did those without AEs. Due to the irreversible deterioration of lung function and new bilateral alveolar infiltrates, these exacerbations can cause the progression of ILD and the occurrence of life-threatening events [31,53,56]. In microscopic polyangiitis-associated ILD, a lower FVC % was an independent prognostic factor for patients to develop AE [54]. Kang et al. [57] reported that a lower DLCO and a radiologic UIP-like pattern at diagnosis were associated with the development of AEs in patients with fibrotic HP. These findings indicate that more attention should be given to PPF patients who have poor PFT and UIP or UIP-like patterns on chest HRCT, especially if the patient experiences AE. We also believe that patients with f-ILD should be considered to have PPF if they have experienced AE.

As a retrospective cohort study, there were still several limitations and deficiencies. First, this was a single-centre study in China with a small sample size, which was representative of only a fraction of the Chinese population. Second, in the process of data collection and follow-up, a small portion of baseline data loss and follow-up bias were inevitable, which may have had an inevitable impact on the statistical results. Finally, all ILD subtypes were included in a single PPF concept, and the impact of various ILD subtypes on the final results was not distinguished. Thus, larger-scale prospective trials based on the different ILD subtypes should be carried out.

## Conclusion

In our study, age >65 years, LDH >245 U/L, CA-153 > 35 U/mL, FVC <60% predicted, DLCO <50% predicted, and the UIP-like pattern on chest HRCT were identified as potential factors associated with the development of PPF in f-ILD populations, and early antifibrotic therapy based on the treatment of the primary disease should be considered. Patients with PPF had poorer outcomes than did those without PPF. Increased SPAP and AE are independent risk factors for the prognosis of PPF patients.

## Data Availability

The data supporting our findings are available from the corresponding author upon reasonable request.
